# Update on PD-1/PD-L1 Inhibitors in Multiple Myeloma

**DOI:** 10.3389/fimmu.2018.02431

**Published:** 2018-11-16

**Authors:** Tomas Jelinek, Bruno Paiva, Roman Hajek

**Affiliations:** ^1^Department of Haematooncology, University Hospital Ostrava, Ostrava, Czechia; ^2^Faculty of Science, University of Ostrava, Ostrava, Czechia; ^3^Faculty of Medicine, University of Ostrava, Ostrava, Czechia; ^4^Centro de Investigación Médica Aplicada, Clínica Universidad de Navarra, IDISNA, Pamplona, Spain

**Keywords:** multiple myeloma, PD-1, PD-L1, pembrolizumab, nivolumab, durvalumab, safety, toxicity

## Abstract

The treatment of cancer, especially of various types of solid tumors, has been revolutionized by the blockade of the PD-1/PD-L1 pathway by immune checkpoint inhibitors. Their success amongst hematologic malignancies, however, has been limited so far to the treatment of classic Hodgkin's lymphoma, which portrays a typical overexpression of PD-1 ligands (PD-L1, PD-L2) as a consequence of changes in chromosome 9p24.1. Their current application in multiple myeloma (MM) is rather uncertain, as discordant results have been reported by distinct research groups concerning especially the expression of PD-1/PD-L1 molecules on malignant plasma cells or on the responsible immune effector cell populations, respectively. In MM it seems that an approach based on combination treatment might be appropriate as unsatisfactory results have been yielded by monotherapy with PD-1/PD-L1 inhibitors. Immunomodulatory drugs, which are the current cornerstone of MM treatment, are the most logical partners as they possess many possibly synergistic effects. Nevertheless, the initially optimistic results have become disappointing due to the excessive and unpredictable toxicity of the combination of pembrolizumab with lenalidomide or pomalidomide. The FDA has suspended or put on hold several phase 3 trials in relapsed as well as in newly diagnosed myeloma patients. There are also other potentially synergistic and promising combinations, such as the anti-CD38 monoclonal antibody daratumumab, irradiation, etc. Not only the effective partner but also the correct timing of the initiation of the PD-1/PD-L1 inhibitors treatment seems to be of utmost importance. These strategies are currently being examined in various stages of myeloma such as during consolidation post autologous stem cell transplantation, targeting minimal residual disease or even in high risk smoldering myeloma.

## Introduction

Multiple myeloma (MM) is a genetically heterogeneous clonal plasma cell disorder which is virtually always preceded by monoclonal gammopathy of undetermined significance (MGUS), an asymptomatic premalignant stage (1, 2). An increased threat of progression to symptomatic disease is represented by smoldering multiple myeloma (SMM), the transitional clinical stage between MGUS and MM (3). MM represents ~1% of all cancers with the estimated incidence of 6 cases per 100,000 persons per year (1, 4, 5) and is the second most common hematologic malignancy. Though still believed to be incurable by many authors, recent progress in its treatment has indicated that the so-called operational cure can be achieved by at least a small proportion of these patients (6). Proteasome inhibitors (PIs) and immunomodulatory drugs (IMiDs) have become the standard of care and newer generations of these agents (pomalidomide, carfilzomib, ixazomib) have been recently approved (7–9). Moreover, there has been recent implementation in routine clinical practice of molecules that possess distinct mechanisms of action like monoclonal antibodies (daratumumab [anti-CD38], elotuzumab [anti-CS1]) and histone deacetylase inhibitors [panobinostat] (10, 11). Daratumumab and isatuximab (both anti-CD38 mAbs) have especially demonstrated exceptional results in relapsed as well as in newly diagnosed myeloma (12–17).

Immunotherapy has proven to be very encouraging in the therapy of many cancers. Its objective is the identification of malignant cells and their annihilation by the process of stimulating and provoking the body's own immune system (18). In MM, immunotherapy efficacy depends on the observation that while allogeneic stem cell transplantation is limited by its toxicity, it is curative for a subset of patients with MM due to the graft-versus-myeloma effect (19). In order to develop new tools to elicit the myeloma-specific immune response, the mAbs targeting surface antigens on malignant plasma cells such as the above-mentioned daratumumab, elotuzumab, isatuximab, and others have been introduced. Cellular therapy, including dendritic cell vaccines, bi-specific mAbs (especially BCMA—T cell bi-specific antibody), chimeric antigen receptor T cells—CAR T cells [the most promising being CAR-T cells targeting BCMA (B cell maturation antigen)] is another form of immunotherapy (20–22). This review aims to describe a group of mAbs targeting immune checkpoints that represent a novel group of immunotherapeutic agents.

## Targeting immune checkpoints

Immune checkpoints, a plethora of inhibitory or stimulatory pathways, are encoded in the immune system and are essential for self-tolerance and also for the modulation of physiological immune responses. The processes of activation, maturation and expansion of T lymphocytes, and inhibition of their apoptosis are supported by stimulatory checkpoints and their ligands (e.g., CD137/CD137L, CD28/CD80, and CD86, CD27/CD70, CD40/CD40L, OX40/OXO4OL, GITR/GITRL, ICOS/ICOSL), while an opposite effect is elicited by inhibitory checkpoints with their ligands (PD-1/PD-L1 and PD-L2, CTLA-4/CD80 and CD86, A2AR/adenosine, KIR/MHC class I, LAG3/MHC class II) (18). They are crucial against the activation of autoimmunity and for the protection against damage of tissue when the immune system is activated against an infection under normal circumstances (23). Nevertheless, there is a possibility that tumor cells may become invisible to the host's immune system when they start to express ligands of checkpoint receptors on their surface and thus abuse and hijack these native pathways (24). Inhibitory immune checkpoint blockade with blocking mAbs (immune checkpoint inhibitors) has consequently emerged as a novel option for cancer treatment. Indeed, checkpoint inhibitors are now a conventional part of the treatment of numerous types of solid tumors (melanoma, non-small cell lung cancer, renal cell carcinoma, head and neck carcinoma) and Hodgkin's lymphoma (25–28).

## PD-1/PD-L1 pathway

There are two chief, well-described inhibitory pathways: (i) cytotoxic T-lymphocyte associated protein 4 (CTLA-4, CD152) as a checkpoint receptor and its cognate ligands B7–1 (CD80) and B7–2 (CD86), and (ii) programmed-death 1 (PD-1, CD279) receptor with its two ligands PD-L1 (CD274, B7-H1) and PD-L2 (CD273, B7-DC). PD-1, a 288 amino acid type I transmembrane protein, is a part of the CD28 receptor family and is expressed on antigen-activated and exhausted T and B cells (29). Two ligands, PD-L1 and PD-L2, are expressed on antigen binding cells (macrophages and dendritic cells) as well as on a subset of activated B lymphocytes and microvascular endothelial cells. Furthermore, there has been a detection of a constitutive level of PD-L1 expression on the cells of various tissues (heart, lung, liver, pancreatic islet cells, astrocytes, etc.) (30, 31). As described earlier, engagement of the PD-1 receptor with its ligands PD-L1 or PD-L2 prompts the temporary down-regulation of T cell function (24, 30, 32). It was recently discovered that not only T cells, but tumor-associated macrophages and NK cells too are involved in the PD-1/PD-L1 pathway (33, 34). Tumor cell visibility for the host immune system is regained by targeting either PD-1 or its receptors (PD-L1/PD-L2) by blocking mAbs, thus leading to the annihilation of the cancer cells.

Many of these checkpoint inhibitors are examined also in MM. This review provides a comprehensive update mainly focused on the clinical efficacy and toxicity of these drugs and their combinations.

## Preclinical data and rationale for combinations

### Expression of PD-L1 on myeloma plasma cells

There are discrepancies between many research groups concerning PD-L1 expression on plasma cells (PCs). It has been demonstrated by several studies that PD-L1 expression is limited to PCs of MM patients, and is absent on those of healthy donors (HD) (35–38). Likewise, PD-L1 expression was reported to be higher on PCs in MM and SMM than in MGUS (35, 39). On the other hand, no differences have been found in the expression of PD-L1 in MM, MGUS and HD by Paiva et al. and Kelly et al. studies (40, 41). The Dhodapkar et al. study has also shown that PD-L1 expression on malignant PCs was associated with an increased risk of progression from SMM to MM (39). Interestingly, the Paiva et al. study has revealed statistically higher PD-L1 expression on clonal PCs from MRD positive MM patients compared to PCs from HD (40).

### Expression of PD-L1 and PD-1 on immune cell subsets

A crucial role in regulating the response of T cells against tumors is played by dendritic cells (DCs). The BM of MM patients was found to have increased levels of plasmocytoid DCs (pDCs). Their diminished ability to trigger T cell response contributed to immune dysfunction (42). The over-expression of PD-L1 on pDCs in MM patients has been demonstrated by several authors (37, 43).

Numerous studies have demonstrated that there is an overexpression of PD-1 on CD4+ and CD8+ T cells from MM patients compared to HD (38, 44). Paiva et al. have shown a significant surge in PD-1 expression on CD4+ and CD8+ T cells only in relapsed or relapsed/refractory MM (RRMM) and MRD positive MM patients (40). PD-1 absence on normal CD56+CD3- NK cells from HD has been confirmed by all published studies. A markedly increased expression of PD-1 on NK cells from MM patients compared to HD was reported by the Benson et al. and Görgün et al. studies; whereas Paiva et al. found no difference between them (34, 38, 40).

### Rationale for the combination of PD-1/PD-L1 inhibitors and IMiDs

Immunomodulatory drugs are back-bone agents in the treatment of newly diagnosed MM (NDMM) as well as in RRMM patients (45). IMiDs, possessing many potentially synergistic properties, could enhance the efficacy of PD-1/PD-L1 blockade. Lenalidomide has been shown to: (i) directly down-regulate PD-L1 expression on MM PCs, (ii) decrease the levels of regulatory T cells (Tregs), (iii) co-stimulate T and NK cells and (iv) down-regulate PD-1 expression on T cells (34, 38, 46–48). This combination, however, has the potential to unleash the immune response, leading to severe toxicity.

The Boston group pioneered preclinical work testing the combination of PD-1/PD-L1 blockade with lenalidomide. FACS sorted T cells and NK cells were separately co-cultured with CD138+ MM cells from RRMM patients in addition to anti-PD-1, anti-PD-L1, alone or in combination, and with lenalidomide. They have demonstrated that effector cell-mediated anti-myeloma cytotoxicity is induced by the blockade of PD-1 and PD-L1 alone, and more significantly, in combination with each other. They found that checkpoint blockade-mediated cytotoxicity is further enhanced by lenalidomide (38).

### Rationale for the combination of PD-1/PD-L1 inhibitors and elotuzumab

There has been recent demonstration that elotuzumab and anti-PD-1 mAb in combination lead to antitumor efficacy enhancement in the myeloma mouse model in the study published by Bezman et al. In these mouse models, combination treatment with elotuzumab and anti–PD-1 promoted tumor-infiltrating NK and CD8^+^ T-cell activation, as well as increased intra-tumoral cytokine and chemokine release. The rationale for the clinical investigation of elotuzumab/anti–PD-1 combination therapy in patients with MM has been supported by these observations (49).

### Rationale for the combination of PD-1/PD-L1 inhibitors and Anti-CD38 mAbs

Anti-CD38 mAbs such as daratumumab or isatuximab are highly effective breakthrough agents for the treatment of MM, with daratumumab already being approved in several indications. As was demonstrated by Chen et al. in the lung cancer mouse model, CD38 could act as a mechanism of resistance in the context of anti-PD-1/PD-L1 therapy. The combination therapy of anti-PD-L1 and anti-CD38 demonstrated a dramatic therapeutic benefit on primary lung cancer tumor growth and metastasis (50). This finding was confirmed also in the MM mouse model J558 by Bezman et al. who demonstrated that combined treatment of anti-CD38 and anti-PD-1 mAb was more effective than each of them alone (51). Thus, based on these preclinical data this combination stands to yield promising results.

### Rationale for the combination of PD-1/PD-L1 inhibitors and irradiation

Radiation therapy (RT), a procedure already renowned for its synergism with checkpoint inhibitors, is a promising candidate for combination treatment due to its capacity for the induction of cancer cell death as well as the mobilization of immune responses for tumor control (52, 53). Radiation appears to intensify cancer cell annihilation and the release of DNA, with a resulting augmentation of T cell priming mediated by dendritic cells. Immune-mediated tumor regression in specific locations out of the irradiated field can be a result of localized RT through the so-called “abscopal effect” (54–56). Preclinical evidence of the efficacy of PD-1/PD-L1 inhibitors plus irradiation is based on the study in the myeloma mouse model. The administration of PD-L1 blockade post lymphodepleting irradiation led to the survival of approximately 66% of mice, compared to 0% in the control group (irradiation without anti-PD-L1 mAb). Interestingly, there was evidence of complete abrogation of the therapeutic efficacy of irradiation plus anti–PD-L1 due to the depletion of either CD4+ or CD8+ T cells. Depletion of NK cells, on the other hand, did not cause any marked effect on therapeutic efficacy (57). This hypothesis has been further confirmed also in the clinical trial phase 1, when only one patient with nivolumab monotherapy reached CR which was only after therapeutic RT on the rib plasmocytoma (58).

### Rationale for the use of PD-1/PD-L1 inhibitors after ASCT

Administration of checkpoint inhibitors after ASCT as consolidation therapy has an immunologic merit. On the basis of the study by Chung et al., it is believed that during this period the Tregs numbers drop, tumor burden reaches nadir, and CD8+ cytototoxic lymphocytes increase in number and express checkpoint inhibitory molecules such as PD-1 and others (59). There is also preclinical evidence of this approach based on the myeloma mouse model when anti-PD-L1 mAbs were administered with cell vaccination. PD-L1 blockade was used after ASCT and administration of whole cell vaccination. This exhibited an improvement in survival from 0 to 40% of myeloma bearing mice in comparison to ASCT and whole cell vaccination alone (60). Indeed, initiation of immunotherapy at this point may be clinically relevant and several studies are already ongoing.

## Clinical data

Monoclonal antibodies targeting the PD-1/PD-L1 axis can be essentially separated into two groups: (i) those against the PD-1 receptor and (ii) those against the ligands (PD-L1/PD-L2). Nivolumab (OPDIVO, MDX1106, BMS-936558, Bristol-Myers Squibb)—a fully human IgG4 mAb; pembrolizumab (KEYTRUDA, MK-3475, Merck)—a highly selective humanized IgG4 mAb, pidilizumab (MDV9300, CT-011, Medivation/Pfizer)—an IgG1 mAb, cemiplimab (REGN-2810, Sanofi), PDR001 (Novartis), and JNJ-63723283 (Janssen) are the main anti-PD-1 mAbs in use. The most promising anti-PD-L1 mAbs are durvalumab (Imfinzi, AstraZeneca), atezolizumab (Tecentriq, Roche), and BMS-936559 (Bristol-Myers Squibb). All available clinical results are summarized in Table 1.

**Table 1:** Available results of clinical trials with PD-1/PD-L1 inhibitors in multiple myeloma.

**Title (author)**	***N***	**Con**.	**T**	**Experimental**	**ORR**	**CR**	**SD**	**Identifier**
			**(*n*)**	**arm**	***n* (%)**	***n* (%)**	***n* (%)**	**Phase**
**PEMBROLIZUMAB**								
A Trial of Pembrolizumab (MK-3475) in Participants With Blood Cancers (MK-3475-013/KEYNOTE-013) (61)	100	RRMM	4	Pembrolizumab	0/30 (0)	0/30 (0)	17/30 (57)	NCT01953692 1
A Study of Pembrolizumab (MK-3475) in Combination With Standard of Care Treatments in Participants With Multiple Myeloma (MK-3475-023/KEYNOTE-023) (62)	115	RRMM	4	Pembrolizumab/ Lenalidomide/ Dexamethasone	20/40 (50)	1/40 (3)	19/40 (48)	NCT02036502 1
1454GCC: Anti-PD-1 (MK-3475) and IMiD (Pomalidomide) Combination Immunotherapy in Relapsed/ Refractory Multiple Myeloma (63)	48	RRMM	3	Pembrolizumab/ Pomalidomide/ Dexamethasone	29/48 (60)	4/48 (8)	14/48 (30)	NCT02289222 1, 2
Pembrolizumab + Lenalidomide Post Autologous Stem Cell Transplant (ASCT) in High-risk Multiple Myeloma (MM) (64)	43	NDMM RRMM	NA	Pembrolizumab/ Lenalidomide/ Dexamethasone	12/12 (100)	1/12 (8)	NA	NCT02906332 2 suspended
Phase 2 Multi-center Study of Anti-PD-1 During Lymphopenic State After HDT/ASCT for Multiple Myeloma (65)	50	NDMM	0	Pembrolizumab/ Lenalidomide	29/29 (100)	7/23(31)	NA	NCT02331368 2
Pembrolizumab (MK-3475) in MM Patients With Residual Disease (66)	20	NDMM RRMM	0 1	Pembrolizumab	3/14 (21)	2/14 (14) sCR 1/14 (7) iCR	5/11 (42)	NCT02636010 2
Study of Lenalidomide and Dexamethasone With or Without Pembrolizumab (MK-3475) in Participants With Newly Diagnosed Treatment Naive Multiple Myeloma (MK-3475-185/KEYNOTE-185) #	640	NDMM	0	a) Lenalidomide/ Dexamethasone b) Pembrolizumab/ Lenalidomide/ Dexamethasone	93/150 (62) 97/151 (64)	NA NA	NA NA	NCT02579863 3 suspended
Study of Pomalidomide and Low Dose Dexamethasone With or Without Pembrolizumab (MK-3475) in Refractory or Relapsed and Refractory Multiple Myeloma (rrMM) (MK-3475-183/KEYNOTE-183) #	300	RRMM	NA	a) Pembrolizumab/ Pomalidomide/ Dexamethasone b) Pomalidomide/ Dexamethasone	50/124 (40) 43/125 (34)	NA NA	NA NA	NCT02576977 3 suspended
**NIVOLUMAB**								
An Investigational Immuno-Therapy Study to Determine the Safety and Effectiveness of Nivolumab and Daratumumab, With or Without Pomalidomide and Dexamethasone, in Patients With Multiple Myeloma (58, 67)	375	RRMM	3 5	Nivolumab Nivolumab/ Ipilimumab	0/27 (0) 0/7 (0)	0/27 (0) 0/7 (0)	17/27 (63) 1/7 (14)	NCT01592370 1 put on hold (enrolment resumed)
Check Point Inhibition After Autologous Stem Cell Transplantation in Patients at High Risk of Post Transplant Recurrence (CPIT001) (68)	42	NDMM RRMM	NA	Nivolumab/ Ipilimumab	NA 4/4 (100)	NA 4/4 (100)	NA 0/4 (0)	NCT02681302 1, 2
**PIDILIZUMAB**								
Lenalidomide and Pidilizumab in Treating Patients With Relapsed/Refractory Multiple Myeloma (69)	53	RRMM	2	Pidilizumab/ Lenalidomide	4/12 (33)	NA	4/12 (33)	NCT02077959 1/2
Blockade of PD-1 in Conjunction With the Dendritic Cell/Myeloma Vaccines Following Stem Cell Transplantation (70)	35	NDMM	0	Pidilizumab Pidilizumab/ Dendritic Cell Fusion Vaccine	NA 12/44 (54)	NA 6/22 (27)	NA NA	NCT01067287 2

Pembrolizumab - mAb anti-PD-1; Nivolumab - mAb anti-PD-1; Pidilizumab - mAb anti-PD-1; mAb - monoclonal antibody; N -estimated enrolment; Con. - condition; RRMM - relapsed or refractory multiple myeloma; NDMM - newly diagnosed multiple myeloma; T - prervious therapies; m - median number of previous therapies; NA, not available; ORR - overall response rate; CR - complete response; sCR - stringent CR; iCR - immunophenotypic CR; SD - stable disease, n - number of assessed patients.

# data were presented on https://www.fda.gov/Drugs/DrugSafety/ucm574305.htm

### Pembrolizumab

#### Pembrolizumab in relapsed or relapsed/refractory multiple myeloma

Monotherapy with pembrolizumab was examined in RRMM patients in the KEYNOTE-013 phase 1b clinical trial. Pembrolizumab at the rate of 10 mg/kg every 2 weeks or at a set dose of 200 mg every 3 weeks in total was administered in 30 patients with a median of 4 previous lines of therapy. None of the subjects experienced response and 57% (17/30) had stable disease (SD). Only one grade 3 adverse event (AE) related to treatment occurred—myalgia (61). At the 2017 EHA (European Hematology Association) meeting, updated preliminary results of the phase 1 study (KEYNOTE-023) of pembrolizumab plus lenalidomide and dexamethasone were presented. In total, 51 RRMM patients with a median of 4 previous lines of therapy received 200 mg of pembrolizumab every 2 weeks, 25 mg of lenalidomide orally on days 1–21, and 40 mg of weekly oral dexamethasone in each 28-day cycle. Responses occurred in 50% (20/40) (1 sCR, 5 VGPR, 14 PR) of patients and there was evidence of progressive disease (PD) in 1 patient. The disease control rate (sCR + CR + VGPR + PR + SD) was 39/40 (98%) in the efficacy population and 28/29 (97%) in the lenalidomide-refractory population with ORR being 38% (11/29) in this subgroup of patients. The most frequent grade ≥3 treatment-related AEs were neutropenia (33%), thrombocytopenia (18%), and anemia (12%); AE related death occurred in 2 patients (4%) (ischemic stroke, hepatic failure). Five (10%) patients suffered from immune-related AEs (irAEs). No incidence of pneumonitis was reported (62). Another combination of pembrolizumab plus pomalidomide and dexamethasone was examined in a single-center phase 2 study. Twenty-eight-day cycles of pembrolizumab 200 mg every 2 weeks, pomalidomide 4 mg daily for 21 days, and dexamethasone 40 mg weekly were administered to 48 RRMM patients with a median of 3 previous lines of therapy. ORR was 60% (29/48), including sCR/CR (8%), VGPR (19%) and PR (33%). Progression-free survival (PFS) was 17.4 months at the median follow-up of 15.6 months, and overall survival (OS) had not yet been reached. Grade 3/4 adverse events occurred in 40% (19/48) of patients. These included pneumonia (15%), hyperglycemia (25%) and hematologic toxicities (40%). Immune-related AEs included pneumonitis (13%) and hypothyroidism (10%), mostly ≤ grade 2 (63). In June 2017, FDA suspended a randomized phase 3 trial (KEYNOTE-183) of pomalidomide and low-dose dexamethasone with or without pembrolizumab in patients with RRMM who had received at least two prior lines of therapy. Two hundred and forty-nine randomized patients were included in a complete evaluation of safety and efficacy of the trial. The ORR in the investigation arm was 34 vs. 40% in the control arm. The median time to progression (TTP) for the pembrolizumab arm was 8.1 vs. 8.7 months in the control arm (HR: 1.14). Median PFS for the pembrolizumab arm was 5.6 vs. 8.4 months in the control arm; HR, 1.53 (95% CI, 1.05–2.22); *P* = 0.98. Median OS was not reached vs. 15.2 months; HR, 1.61 (95% CI, 0.91–2.85); *P* = 0.95 (71). The toxicity issue has particularly been addressed in a specific paragraph of this manuscript^1^

#### Pembrolizumab as consolidation after autologous stem cell transplantation

Pembrolizumab has also been tested as a part of consolidation strategy during the lymphodepleted state post autologous stem cell transplantation (ASCT) for the eradication of the residual clone of plasma cells. First, pembrolizumab monotherapy [200 mg every 3 weeks starting day +14 after ASCT (upon engraftment) for 9 doses (day+180)] was examined in a two-site, single arm phase 2 study. Twenty-nine patients who had not reached CR after induction treatment were enrolled in the study, and the CR rate in the evaluable patients was 31% (7/23). Among 29 patients, there were two grade 3 toxicities (colitis, infusion reaction), and one grade 2 toxicity (radiculopathy) which led to treatment discontinuation. Overall, another 12 irAEs not leading to discontinuation included grade 1–2 events including 4 cases of infusion reactions, 2 cases of hypothyroidism, skin rashes and colitis each and grade 3 events of acute kidney injury and hepatitis. The authors concluded that the administration of pembrolizumab after ASCT was safe. Nevertheless, the results must be interpreted carefully, as it is not possible to distinguish between the effect of HD melphalan and pembrolizumab (65). Another phase 2, single center study was performed in high risk MM patients during the 3–6 months after ASCT. Patients were administered pembrolizumab 200 mg on day 1; lenalidomide 25 mg daily on days 1–14; and dexamethasone 40 mg daily on days 1, 8 and, 15 of a 21-day cycle for a total of 2 cycles and then an additional 2 cycles of pembrolizumab + lenalidomide without corticosteroids at the same dose and frequency. ORR to upfront therapy was 100% with 1 (8.3%) achieving CR, 5 (41.6%) achieving VGPR and 6 (50%) achieving PR to induction. Four patients (33%) achieved stringent CR after the study treatment. 2 patients suffered from non-hematologic grade 3 AEs including hypoxia and maculopapular rash. This study was suspended as of 7th May 2017 after the FDA placed Merck studies using the combination of pembrolizumab and IMiDs on hold (64).

GEM-Pembresid is a Spanish phase 2 clinical trial evaluating pembrolizumab monotherapy as consolidation after ASCT in those patients that achieved at least VGPR but with persistent residual disease. A dose of 200 mg of pembrolizumab was given every 3 weeks for 12 months. Amongst the 14 patients that were evaluable, 3 (21%) upgraded their response, 2 patients in VGPR converted into sCR, and 1 CR patient achieved MRD negativity. There was ongoing reduction of the FLC and MRD levels, respectively, in two additional patients. Treatment with pembrolizumab showed good tolerance with no related AEs. Another objective of the study was to identify biological markers of response or resistance to pembrolizumab. Flow cytometric studies revealed that early progressions were related to lower basal NK numbers and a lower PD1 expression in effector memory CD8+ T cells (66).

#### Pembrolizumab in newly diagnosed multiple myeloma

KEYNOTE-185 was a phase 3, randomized, clinical trial of lenalidomide and low-dose dexamethasone with or without pembrolizumab in patients with NDMM who were ineligible for ASCT. The safety and efficacy analysis included 301 randomized patients. Based on this evaluation, FDA suspended this trial in June 2017 due to excessive toxicity that is further discussed in detail in a specific paragraph of this manuscript. In the investigation arm, the ORR was 64% compared to 62% in the control arm; median TTP was not reached in both arms^2^ Median PFS was not reached in either arm; HR, 1.22 (95% CI, 0.67–2.22); *P* = 0.75 as well as median OS; HR, 2.06 (95% CI, 0.93–4.55); *P* = 0.97 (72). The toxicity issue has particularly been addressed in a specific paragraph of this manuscript.

#### Pembrolizumab in smoldering multiple myeloma

At the ASH (American Society of Hematology) 2017 annual meeting, the preliminary results of a pilot study of pembrolizumab for immunoprevention in smoldering MM were presented. The study included patients with intermediate—high risk smoldering multiple myeloma (I-HR-SMM) according to the PETHEMA, Mayo, or SWOG criteria. Pembrolizumab doses were given at 200 mg every 21 days for up to 8 cycles. Those patients that achieved ≥ minor response after 8 cycles were eligible to continue treatment for up to 24 cycles. The target ORR was 25%. Twelve patients with I-HR-SMM were enrolled. Stringent CR was achieved by one patient (8%), 10 patients had SD (83%), and one patient had PD (8%). Therapy had to be discontinued in five patients as a result of related AEs due to elevated liver function tests (*n* = 2), acute kidney injury (*n* = 2), and myalgia (*n* = 1) (73).

Ongoing clinical trials with pembrolizumab are summarized in Table 3.

### Nivolumab

#### Nivolumab in relapsed or relapsed/refractory multiple myeloma

Leshokin et al. have published the results of a phase 1 clinical trial assessing nivolumab as a single-agent in patients with relapsed or refractory T- or B-cell lymphoma or MM. Of the 27 RRMM patients evaluated (median of 3 previous lines of therapy), 63% (17/27) had reached SD as a best response with the exception of one patient who reached CR but only after irradiation of a focal plasmacytoma. Nivolumab's safety profile was similar to that observed in solid tumors. Thirty-four percentage of patients suffered from irAEs, with pneumonitis being the most frequent (11%) (58). The phase 1 study's preliminary results of nivolumab plus ipilimumab were presented at ASH 2016. There was no response in any of the 7 enrolled RRMM patients (with a median of 5 previous therapies), and 14% (1/7) had SD (67).

#### Nivolumab as consolidation after autologous stem cell transplantation

At the 2017 ASH meeting, the initial safety and efficacy data for the combination of nivolumab and ipilimumab as consolidation following ASCT for high-risk hematologic malignancies were presented. 25 patients with different diagnoses including diffuse large B cell lymphoma and peripheral T cell lymphoma were enrolled in the study. 11 MM patients (7 newly diagnosed, 4 relapsed after the first ASCT within 3 years) were also enrolled in the study in total. All 4 relapsed patients (100%) were in sCR after consolidation (before ASCT only 2 had been in sCR). There have been a significant number of irAEs (80%). The nivolumab plus ipilimumab combination was discontinued after six patients (24% total: colitis 12%, pneumonitis 4%, adrenal crisis 4%, and hepatotoxicity 4%) presented with AEs of any grade related to treatment. One case of death that could be attributed to experimental treatment occurred (due to recurrent pneumonitis complicated by parainfluenza) (68).

Table 2 summarizes ongoing clinical trials with nivolumab and other anti-PD-1 mAbs.

**Table 2:** Ongoing clinical trials with Nivolumab and other anti-PD-1 monoclonal antibodies (PDR001, JNJ-63723283, Cemiplimab) in multiple myeloma.

**Title**	***N***	**Con**.	**Experimental arm**	**Identifier Phase**
Nivolumab Role in the Treatment of Patients With Refractory or Relapse Multiple Myeloma	40	RRMM	a) Nivolumab/Pomalidomide/ Dexamethasone b) Nivolumab/Elotuzumab/ Pomalidomide/Dexamethasone	NCT03023527 1 put on hold (enrolment resumed)
An Investigational Immuno-Therapy Study to Determine the Safety and Effectiveness of Nivolumab and Daratumumab, With or Without Pomalidomide and Dexamethasone, in Patients With Multiple Myeloma	375	RRMM	a) Nivolumab b) Nivolumab/Ipilimumab ^§^ c) Nivolumab/Daratumumab d) Nivolumab/Daratumumab/ Pomalidomide/Dexamethasone	NCT01592370 1 put on hold (enrolment resumed)
ASCT With Nivolumab in Patients With Multiple Myeloma	30	NDMM^*^	Nivolumab	NCT03292263 1/2
An Exploratory Study to Evaluate the Combination of Elotuzumab and Nivolumab With and Without Pomalidomide in Relapsed Refractory Multiple Myeloma	70	RRMM	a) Nivolumab/Elotuzumab b) Nivoluma/Elotuzumab/ Pomalidomide/Dexamethasone	NCT03227432 2
A Study of Elotuzumab in Combination With Pomalidomide and Low Dose Dexamethasone and Elotuzumab in Combination With Nivolumabin Patients With Multiple Myeloma Relapsed or Refractory to Prior Treatment With Lenalidomide	95	RRMM	a) Elotuzumab/Pomalidamide/Dexamethasone b) Nivolumab/Elotuzumab	NCT02612779 2 put on hold (enrolment resumed)
Nivolumab Combined With Daratumumab With or Without Lenalidomide	60	RRMM	a) Nivolumab/Daratumumab b) Nivolumab/Daratumumab/ Lenalidomide/Dexamethasone	NCT03184194 2
An Investigational Immuno-therapy Study of Nivolumab, Elotuzumab, Pomalidomide and Dexamethasone Combinations in Patients With Multiple Myeloma (CheckMate 602)	406	RRMM	a) Nivolumab/ Pomalidomide/Dexamethasone b) Pomalidomide/ Dexamethasone c) Nivolumab/Elotuzumab/ Pomalidomide/Dexamethasone	NCT02726581 3 put on hold
Study of Single Agent CJM112, and PDR001 in Combination With LCL161 or CJM112 in Patients With Multiple Myeloma	70	RRMM	a) CJM112 b) PDR001/ CJM112 c) PDR001/LCL161	NCT03111992 1
A Study of JNJ-63723283, an Anti-programmed Death-1 Monoclonal Antibody, Administered in Combination With Daratumumab, Compared With Daratumumab Alone in Participants With Relapsed or Refractory Multiple Myeloma	386	RRMM	a) Daratumumab b) JNJ-63723283/Daratumumab	NCT03357952 1
Isatuximab in Combination With Cemiplimab in Relapsed/Refractory Multiple Myeloma (RRMM) Patients	105	RRMM	Cemiplimab/Isatuximab	NCT03194867 1/2

N - estimated enrolment; Con. - condition; RRMM - relapsed or refractory multiple myeloma; NDMM - newly diagnosed multiple myeloma; mAb - monoclonal antibody; PDR001; mAb anti-PD1 - JNJ-63723283 - mAb anti-PD1; Cemiplimab - mAb anti-PD1; Ipilimumab - mAb anti-CTLA-4; Lirilumab - mAb anti-KIR; Elotuzumab - mAb anti-SLAMF7; CJM112 - mAb anti-IL-17A; LCL161 - mitochondrial-derived activator of caspases mimetic and inhibitor of apoptosis antagonists

* *NDMM who achieved partial remission, stable disease or progression disease after autologous stem cell transplantation*,

§ *Ipilimumab or Lirilumabb*.

### Pidilizumab

#### Pidilizumab in relapsed or relapsed/refractory multiple myeloma

The initial results of the phase 1/2 study of pidilizumab with lenalidomide in RRMM patients were presented at ASH 2015. Of the 12 patients that were evaluable (median of 2 prior lines of therapy), 33% (4/12) responded/had responses and another 33% of patients reached SD (69).

#### Pidilizumab as consolidation after autologous stem cell transplantation

The combination of pidilizumab with a dendritic cell/myeloma fusion cell vaccination was administered post ASCT. Of the 22 RRMM patients that were enrolled, VGPR was reached by 27% (6/22) and CR was reached by another 27% (6/22). We must interpret these results carefully, however, as the type of treatment that led to these outcomes is not clear (70).

## Immune-related toxicity

The therapeutic usage of mAbs such as PD-1 or CTLA-4 that block inhibitory checkpoint molecules may serve to enhance the specific (dominantly T cell) immune response which activates the immune system against the tumor (74). Functional disruption of immune checkpoint molecules, however, can lead to immunologic tolerance imbalances and thus an uncontrolled immune response, which may present clinically with autoimmune-like/inflammatory side-effects, leading to collateral damage of normal tissues and organ systems. Such adverse events are termed ‘immune-related adverse events' (irAEs) and are thought to be principally T-cell mediated (75, 76). The safety data comes dominantly from the studies performed in solid oncology. IrAEs generally occur quite early. They mostly present within weeks to 3 months after the initiation of treatment with immune checkpoint blockers. The most commonly reported AE with anti-PD-1/PD-L1 is fatigue. Across monotherapy studies the incidence of fatigue is 16–37% for anti-PD-1 and 12–24% for anti-PD-L1 (76). The most frequent and typical organ specific irAEs are: (i) dermatologic toxicities, (ii) diarrhea/colitis, (iii) endocrine toxicities, (iv) hepatic toxicities, (v) pneumonitis, and (vi) rare toxicities such as neurologic syndromes, renal toxicity, myocarditis, and others. Standard treatment algorithms for irAEs utilizing immune-modulating medications that include high-dose corticosteroids, antihistamines, anti-tumor necrosis factor α (TNFα) mAbs, and calcineurin inhibitors have been developed (76–78).

Pembrolizumab and nivolumab monotherapy in RRMM patients exhibited safety profiles which were consistent with those observed in other cancers (58, 61). However, concerns have recently been raised regarding the excessive toxicity of the combination of pembrolizumab with IMiDs used specifically in myeloma trials. A move based on safety concerns identified in KEYNOTE-183 and KEYNOTE-185 was made by the FDA. Pembrolizumab in combination with dexamethasone and pomalidomide or lenalidomide for the treatment of RRMM or NDMM patients, respectively, was evaluated by two phase three clinical trials. The discontinuation of both trials was directed by the agency on July 3rd 2017, as according to interim results, an added risk of death was linked to pembrolizumab. In KEYNOTE-183 (*N* = 249, pembrolizumab, pomalidomide, dexamethasone) there were 29 deaths in the pembrolizumab arm vs. 21 deaths in the control arm at the median follow-up of 8.1 months. In the pembrolizumab group the hazard ratio (HR) for overall survival (OS) compared with the control arm was 1.61 (95% CI, 0.91–2.85), meaning an increase of >50% in the relative risk of death. Severe grade 3–5 toxicity was increased by 18% (83 vs. 65%, investigational vs. control arm). The incidence of serious AEs was 63% compared to 46% in the control arm. In the pembrolizumab arm the following non-disease progression causes of death were identifiable: myocarditis, Stevens-Johnson syndrome, myocardial infarction, pericardial hemorrhage, cardiac failure, respiratory tract infection, neutropenic sepsis, sepsis, multiple organ dysfunction, respiratory failure, and unknown (71, 79).

The KEYNOTE-185 (pembrolizumab, lenalidomide, dexamethasone) safety and efficacy analysis included 301 patients. Nineteen deaths were reported at the median follow-up of 6.6 months (HR for OS, 2.06; 95% CI, 0.93–4.55) in the pembrolizumab group compared with 9 in the control arm. The relative risk of death in the pembrolizumab arm was more than double the risk in the control group, the safety analysis saw a 22% increase of severe, grade 3–5 toxicity (72 vs. 50%, investigational vs. control arm) and there was an incidence of 54% of serious AEs compared to 39% in the control arm. The following causes of death, not related to disease progression, were identifiable in the pembrolizumab arm: intestinal ischemia, cardio-respiratory arrest, suicide, pulmonary embolism, cardiac arrest, pneumonia, sudden death, myocarditis, large intestine perforation, and cardiac failure (72, 79)^3^ The discrepancy between positive phase two trials with no safety signals and suspended phase 3 trials is provoking. It may be partially explained by the imbalance between the investigational and control arm at least in the Keynote-185 study. The investigators probed baseline features of patients who had died: age over 80 (42 vs. 33% in the control arm), ISS III disease (31.6 vs. 22.2%), renal impairment (10.5 vs. 0%), hypercalcemia at presentation (21 vs. 11%), and high-risk cytogenetics (26.3 vs. 0%) were more prevalent in the pembrolizumab arm as was stated by Dr. Usmani^4^.

In September 2017 the FDA placed on partial holds three clinical trials that assessed nivolumab-based combinations (the phase 3 CheckMate-602, phase 1 CheckMate-039, and phase 2 CA204142 trials) in patients with RRMM. At the same time, the agency also put a full hold on MEDI4736-MM-002, a phase 1b study which had the aim of establishing an appropriate dose and regimen for the durvalumab and lenalidomide combination with and without low-dose dexamethasone in NDMM patients, as well as on MM-005, a phase 2 study evaluating the combination of durvalumab and daratumumab in RRMM patients.^5^ Analogically two phase 1/2 trials with atezolizumab and lenalidomide or pomalidomide in RRMM patients were put on partial clinical hold at this time-point. Nevertheless, several studies, mainly with nivolumab and atezolizumab, were resumed in December 2017 after a successful safety review observed no increased toxicity. All trials that were suspended or put on hold by FDA are summarized in Table 5.

Recently, an alarming case report describing lethal fulminant myocarditis after a single pembrolizumab dose in a newly diagnosed myeloma patient enrolled in the Keynote-185 trial has been published. The authors also discuss the role of pre-existing occult autoimmunity that may have played a part in such a severe and rapid course of myocarditis leading to death within a few days (80).

The combination of pembrolizumab with IMiDs seems to be toxic indeed and immune-related AEs are severe and unpredictable.

## Conclusion and future perspectives

The PD-1/PD-L1 axis blockade in MM represents a “hot topic,” as there are plenty of ongoing clinical trials summarized in Tables 1–4. Single agent PD-1 blockade is not effective in MM and does not induce any responses in contrast to many solid tumors and Hodgkin's lymphoma (58, 61). It may be partially explained by senescent rather than exhausted phenotype of T cells in MM, thus the PD-1 blockade is not able to re-invigorate their function (81). A combination-based approach is needed and IMiDs as backbone agents in MM possess many potentially synergistic properties (52). Promising results of the pembrolizumab plus either lenalidomide or pomalidomide and dexamethasone combination in heavily pretreated RRMM patients have been recently reported, reaching ORR in about 50–60% (62, 63). However, safety concerns have been raised regarding this combination and FDA suspended two phase 3 trials with pembrolizumab (Keynote-183, Keynote-185) in June 2017. Based on this analysis many other trials including any PD-1/PD-L1 inhibitors in combination with IMiDs have been put on hold, but several of them, especially with nivolumab and atezolizumab, have been restarted after the safety review. Immune-related toxicity is severe and unpredictable. Indeed, from the clinical point of view, this unfavorable toxic profile makes the position of PD-1/PD-L1 inhibitors in frontline treatment or even in smoldering myeloma questionable. Another interesting strategy is to administer anti-PD-1/PD-L1 mAbs after ASCT as a part of consolidation as it also has an immunological merit. Further investigation and randomized trials are needed to prove the effectiveness of this approach. There are many efforts to combine checkpoint inhibitors with other agents or procedures. The most promising seem to be: (i) mAbs targeting surface antigens such as daratumumab or elotuzumab (49), (ii) irradiation because of its abscopal effect and many others that are still under investigation (56, 82). Not only the right partner for the combination but also the right timing of the initiation of treatment seems to be of utmost importance. Finally, the checkpoint inhibitors possess very distinct toxicity profiles from the routinely used agents in MM and thus physicians should be aware of these immune-related adverse events and of the management of these sometimes very complicated situations as well. Either way, blockade of PD-1/PD-L1 pathway may still be a hope for a specific subset of myeloma patients because of its capacity to induce durable responses where other treatment strategies have failed.

**Table 3:** Ongoing clinical trials with Pembrolizumab in multiple myeloma including smoldering multiple myeloma.

**Title**	***N***	**Con**.	**Experimental arm**	**Identifier Phase**
Pembrolizumab for Smoldering Multiple Myeloma (SMM)	16	SMM	Pembrolizumab	NCT02603887 1
NY-ESO-1^*c*259^T Alone and in Combination With Pembrolizumab for Multiple Myeloma	20	RRMM	a) NY-ESO-1^*c*259^T cells/Pembrolizumab b) NY-ESO-1^*c*259^T cells	NCT03168438 1
A Study of Pembrolizumab (MK-3475) in Combination With Standard of Care Treatments in Participants With Multiple Myeloma (MK-3475-023/KEYNOTE-023)	84	RRMM	a) Pembrolizumab/Lenalidomide/Dexamethasone b) Pembrolizumab/Carfilzomib/Dexamethasone	NCT02036502 1
Pembrolizumab and Radiation Therapy in Patients With Relapsed or Refractory Multiple Myeloma	24	RRMM	Pembrolizumab/RT	NCT03267888 1
ACP-196 (Acalabrutinib) in Combination With Pembrolizumab, for Treatment of Hematologic Malignancies (KEYNOTE145)	159	RRMM	Pembrolizumab/Acalabrutinib	NCT02362035 1, 2
Efficacy and Safety Study of Pembrolizumab (MK-3475) in Combination With Daratumumab in Participants With Relapsed Refractory Multiple Myeloma (MK-3475-668/KEYNOTE-668)	57	RRMM	Pembrolizumab/Daratumumab	NCT03221634 2
Pembrolizumab, Ixazomib Citrate, and Dexamethasone in Treating Participants With Relapsed Multiple Myeloma	42	RRMM	Pembrolizumab/Ixazomib/Dexamethasone	NCT03506360 2

*N - estimated enrolment; Con. - condition; RRMM - relapsed or refractory multiple myeloma; SMM - smoldering myeloma; Pembrolizumab - mAb anti-PD-1; Acalabrutinib - Bruton's tyrosine kinase inhibitor; NY-ESO-1^c259^T cells - autologous genetically modified T Cells*.

**Table 4:** Ongoing clinical trials with PD-L1 inhibitors in multiple myeloma (Atezolizumab, Durvalumab, BMS-936559).

**Title**	***N***	**Con**.	**Experimental arm**	**Identifier Phase**
**ATEZOLIZUMAB**				
Study of Atezolizumab (Anti-Programmed Death-Ligand 1 [PD-L1] Antibody) Alone or in Combination With an Immunomodulatory Drug and/or Daratumumab in Participants With Multiple Myeloma (MM)	288	RRMM	a) Atezolizumab b) c^*^) Atezolizumab/Lenalidomide d) Atezolizumab/Daratumumab e) Atezolizumab/Daratumumab/Lenalidomide f) Atezolizumab/Daratumumab/Pomalidomide	NCT02431208 1 put on hold (enrolment resumed)
Pilot Study Of Anti-Programmed Death Ligand-1 (Anti-PD-L1, Atezolizumab) In Asymptomatic Myeloma	20	SMM	Atezolizumab	NCT02784483 1 suspended
A Study of Cobimetinib Administered as Single Agent and in Combination With Venetoclax, With or Without Atezolizumab, in Participants With Relapsed and Refractory Multiple Myeloma	72	RRMM	a) Atezolizumab/Cobimetinib b) Cobimetinib/Venetoclax c) Atezolizumab/Cobimetinib/Venetoclax	NCT03312530 1, 2
**DURVALUMAB**				
A Study to Determine Dose and Regimen of Durvalumab as Monotherapy or in Combination With Pomalidomide With or Without Dexamethasone in Subjects With Relapsed and Refractory Multiple Myeloma	138	RRMM	a) Durvalumab b) Durvalumab/Pomalidomide c) Durvalumab/Pomalidomide/Dexamethasone	NCT02616640 1 put on hold
A Study of Durvalumab in Combination With Lenalidomide With and Without Dexamethasone in Subjects With Newly Diagnosed Multiple Myeloma	138	NDMM	a) Durvalumab/Lenalidomide b) Durvalumab/Lenalidomide/ Dexamethasone	NCT02685826 1 suspended
A Study of PVX-410, a Cancer Vaccine, and Durvalumab +/- Lenalidomide for Smoldering MM	26	SMM	a) Durvalumab b) Durvalumab/PVX-410 c) Durvalumab/PVX-410/Lenalidomide	NCT02886065 1
Phase 1 Study to Assess Safety & Tolerability of Tremelimumab & Durvalumab, Administered With High Dose Chemotherapy and Autologous Stem Cell Transplant	24	RRMM^§^	Durvalumab/Tremelimumab	NCT02716805 1suspended
A Study to Determine the Safety and Efficacy for the Combination of Durvalumab and Daratumumab in Relapsed and Refractory Multiple Myeloma (FUSIONMM-003)	144	RRMM	a) Durvalumab/Daratumumab b) Durvalumab/Daratumumab/ Pomalidomid/Dexamethasone	NCT02807454 2 put on hold
A Study to Determine the Efficacy of the Combination of Daratumumab (DARA) Plus Durvalumab (DURVA) (D2) in Subjects With Relapsed and Refractory Multiple Myeloma (RRMM) (FUSION-MM-005)	180	RRMM	Durvalumab/Daratumumab	NCT03000452 2 suspended
**BMS-936559**				
Safety Study of Anti-Programmed Death-Ligand 1 in Hematologic Malignancy	110	RRMM	BMS-936559	NCT01452334 1 withdrawn

*RRMM - relapsed or refractory multiple myeloma; NDMM - newly diagnosed multiple myeloma; SMM - smoldering multiple myeloma; N - estimated enrolment; Con.- condition; mAb - monoclonal antibody; autoHSCT - autologous stem cell transplantation; Atezolizumab - mAb anti-PD-L1; Durvalumab - mAb anti-PD-L1; BMS-936559 - mAb anti-PD-L1; Tremelimumab - mAb anti-CTLA-4; PVX-410, tetra-peptide vaccine against XBP1, CD138, and CS1*,

* *Atezolizumab/Lenalidomide is administrated to patients who have measurable disease after autoHSCT*,

§ *Tremelimumab or Tremelimumab/Durvalumab is administrated prior to and for 2 cycles post autoHSCT followed by up to 6 additional monthly cycles of durvalumab alone*.

**Table 5:** Suspended and put on hold clinical trials with PD-1/PD-L1 inhibitors in multiple myeloma.

**Title**	***N***	**Con**.	**Experimental arm**	**Identifier Phase**
Pembrolizumab + Lenalidomide Post Autologous Stem Cell Transplant (ASCT) in High-risk Multiple Myeloma (MM)	43	NDMM RRMM	Pembrolizumab/Lenalidomide/Dexamethasone	NCT02906332 2 suspended
Study of Lenalidomide and Dexamethasone With or Without Pembrolizumab (MK-3475) in Participants With Newly Diagnosed Treatment Naive Multiple Myeloma (MK-3475-185/KEYNOTE-185)	640	NDMM	a) Lenalidomide/Dexamethasone b) Pembrolizumab/Lenalidomide/Dexamethasone	NCT02579863 3 suspended
Study of Pomalidomide and Low Dose Dexamethasone With or Without Pembrolizumab (MK-3475) in Refractory or Relapsed and Refractory Multiple Myeloma (rrMM) (MK-3475-183/KEYNOTE-183)	300	RRMM	a) Pembrolizumab/Pomalidomide/Dexamethasone b) Pomalidomide/Dexamethasone	NCT02576977 3 suspended
An Investigational Immuno-Therapy Study to Determine the Safety and Effectiveness of Nivolumab and Daratumumab, With or Without Pomalidomide and Dexamethasone, in Patients With Multiple Myeloma	375	RRMM	a) Nivolumab b) Nivolumab/Ipilimumab^§^ c) Nivolumab/Daratumumab d) Nivolumab/Daratumumab/ Pomalidomide/Dexamethasone	NCT01592370 1 put on hold (enrolment resumed)
A Study of Elotuzumab in Combination With Pomalidomide and Low Dose Dexamethasone and Elotuzumab in Combination With Nivolumabin Patients With Multiple Myeloma Relapsed or Refractory to Prior Treatment With Lenalidomide	95	RRMM	a) Elotuzumab/Pomalidamide/Dexamethasone b) Nivolumab/Elotuzumab	NCT02612779 2 put on hold (enrolment resumed)
An Investigational Immuno-therapy Study of Nivolumab, Elotuzumab, Pomalidomide and Dexamethasone Combinations in Patients With Multiple Myeloma (CheckMate 602)	406	RRMM	a) Nivolumab/Pomalidomide/Dexamethasone b) Pomalidomide/Dexamethasone c) Nivolumab/Elotuzumab/ Pomalidomide/Dexamethasone	NCT02726581 3 put on hold
Study of Atezolizumab (Anti-Programmed Death-Ligand 1 [PD-L1] Antibody) Alone or in Combination With an Immunomodulatory Drug and/or Daratumumab in Participants With Multiple Myeloma (MM)	288	RRMM	a) Atezolizumab b) c*) Atezolizumab/Lenalidomide d) Atezolizumab/Daratumumab e) Atezolizumab/Daratumumab/Lenalidomide f) Atezolizumab/Daratumumab/Pomalidomide	NCT02431208 1 put on hold (enrolment resumed)
Pilot Study Of Anti-Programmed Death Ligand-1 (Anti-PD-L1, Atezolizumab) In Asymptomatic Myeloma	20	SMM	Atezolizumab	NCT02784483 1 suspended
A Study to Determine Dose and Regimen of Durvalumab as Monotherapy or in Combination With Pomalidomide With or Without Dexamethasone in Subjects With Relapsed and Refractory Multiple Myeloma	138	RRMM	a) Durvalumab b) Durvalumab/Pomalidomide c) Durvalumab/Pomalidomide/Dexamethasone	NCT02616640 1 put on hold
A Study of Durvalumab in Combination With Lenalidomide With and Without Dexamethasone in Subjects With Newly Diagnosed Multiple Myeloma	138	NDMM	a) Durvalumab/Lenalidomide b) Durvalumab/Lenalidomide/Dexamethasone	NCT02685826 1 suspended
A Study to Determine the Safety and Efficacy for the Combination of Durvalumab and Daratumumab in Relapsed and Refractory Multiple Myeloma (FUSIONMM-003)	144	RRMM	a) Durvalumab/Daratumumab b) Durvalumab/Daratumumab/ Pomalidomid/Dexamethasone	NCT02807454 2 put on hold
A Study to Determine the Efficacy of the Combination of Daratumumab (DARA) Plus Durvalumab (DURVA) (D2) in Subjects With Relapsed and Refractory Multiple Myeloma (RRMM) (FUSION-MM-005)	180	RRMM	Durvalumab/Daratumumab	NCT03000452 2 suspended

*N - estimated enrolment; Con. - condition; RRMM - relapsed or refractory multiple myeloma; NDMM - newly diagnosed multiple myeloma; SMM - smoldering myeloma; mAb - monoclonal antibody; Pembrolizumab - mAb anti-PD-1; Pidilizumab - mAb anti-PD-1; Nivolumab - mAb anti-PD-1; Atezolizumab - mAb anti-PD-L1; Durvalumab - mAb anti-PD-L1*.

## Author contributions

TJ wrote the manuscript, concept, work coordination. BP and RH overall proofread.

### Conflict of interest statement

The authors declare that the research was conducted in the absence of any commercial or financial relationships that could be construed as a potential conflict of interest.

## Footnotes

^1^https://www.fda.gov/Drugs/DrugSafety/ucm574305.htm

^2^https://www.fda.gov/Drugs/DrugSafety/ucm574305.htm

^3^https://www.fda.gov/Drugs/DrugSafety/ucm574305.htm

^4^https://am.asco.org/analyses-data-halted-keynote-trials-presented-poster-sessions

^5^https://www.onclive.com/web-exclusives/fda-places-holds-on-several-durvalumab-combination-trials
